# Evaluation of the Effect of Tooth Type and Canal Configuration on Crown Size in Mandibular Premolars by Cone-Beam Computed Tomography

**Published:** 2013-10-07

**Authors:** Mohammad SalarPour, Narges Farhad Mollashahi, Elnaz Mousavi, Elahe SalarPour

**Affiliations:** a Department of Endodontics, Dental school, Zahedan University of Medical Sciences and Health Services, Zahedan, Iran

**Keywords:** Crown Size, Cone-Beam Computed Tomography (CBCT), Root Canal Morphology, Tooth Crown

## Abstract

**Introduction:**

To achieve success in treatment, one cannot ignore the knowledge of pulp anatomy. Mandibular premolars are considered to be the most difficult teeth for endodontic therapy due to high variability in their canal morphology. It is possible that a relation exists between the crown size and the number of extra canals in these teeth, so this *in vitro* study aims to investigate the relationship between the crown size and the uncommon morphology of mandibular premolars using Cone-Beam Computed Tomography (CBCT).

**Materials and Methods:**

Eighty three extracted mandibular human premolars were exposed to radiation using the CBCT device. Root canal configuration was categorized according to the Vertucci’s classification. The crown size was measured in three axial, coronal and sagittal sections. Finally, the relation between these two factors was evaluated with variance analysis (two-way ANOVA) and chi-square.

**Results:**

The most common canal type in the mandibular first and second premolars are type I (71% and 76%, respectively), followed by type V (29% and 22%, respectively). No significant relationship was found between the crown size and extra canals in mandibular premolars (*P*>0.05).

**Conclusion:**

In this *in vitro* study, the average crown size in two-canalled second premolars was less than that in first premolars with a single canal; although the difference was not statistically significant. The research hypothesis was therefore rejected in both first and second mandibular premolars.

## Introduction

In order to achieve success in endodontic treatment, the knowledge of pulp anatomy could not be ignored. Undetected canals are the cause of 42% of root canal retreatments[1]. Mandibular first premolars are known as the most difficult teeth to treat in endodontics, and have the highest rate of non-surgical endodontic treatment failure (11.45%); the reason is attributed to the wide variety of root canal morphology and difficult access to the second canal [2, 3]. On the other hand, there is a high incidence of mandibular premolars with more than one canal; prevalence of two canals in first and second premolars is 27.8% and 8.9%, respectively, and this will affect the outcomes [2].

Unfortunately, the two-canal morphology of the mandibular premolars is rarely considered in diagnostic radiography. The lingual inclination of the crown towards the root, specifically in the first premolar, and also, the separation of the secondary canal with an acute angle, leads to the second canal remaining undiagnosed both in radiography and tactile examinations [4].

Modifying the horizontal angle of radiography, paying attention to disappearance or rapid narrowing of the main canal in radiography (fast break), and meticulous searching with file tip usually facilitate the discovery of the second canal for clinicians [5].

In a case report, Nallapati declared that in mandibular premolars with more than one canal, the cervical half of the root is often wider than usual with or without a low taper. Therefore, an accurate interpretation of the crown and root morphology of these teeth could be a sign of extra canals [1]. In a similar study, Warren and Laws [6] investigated the

relationship between the crown size and the prevalence of two root canals in mandibular incisors by Peck & Peck index of orthodontics. This index describes the numerical expression of the crown shape and is the result of dividing maximum mesiodistal (MD) diameter by maximum faciolingual (FL) diameter multiplied by 100. Using caliper, they calculated Peck & Peck index for teeth determined as two-canal incisors in radiography, and evaluated the relationship between the teeth with two canals and the index [6].

Today, some progress has been made in producing three-dimensional images, among which, Cone-beam Computed Tomography (CBCT) technology provides us with some information concerning extra canals, apical deltas, canal type, accurate measurement potential in all aspects of root canal system, and in total, a three-dimensional image of root canal(s) anatomy [7, 8].

Therefore, according to the complexities in treating mandibular premolars, high prevalence of extra canals in these teeth and its possible relationship with crown size, and considering the high potential of CBCT in diagnosis of such cases, we evaluated the relationship between the crown size (as an indicator) and root canal morphology in mandibular premolars by means of a CBCT device in this *in vitro* study.

## Material and Methods

The plan was carried out after approving by the Scientific Committee (No.91-1533) and observing moral codes adopted by the National Ethics Committee in Medical Sciences Research.

The present study was conducted on 84 extracted mandibular premolars in two groups with equal numbers of teeth. The selected teeth had complete roots and healthy external morphologies. The excluded teeth were: those with very narrow, blocked canals or other defects such as internal and external root resorption, as well as two-rooted premolars. Also the endodontically treated teeth and those with multiple decays encompassing much of the crown and root structure so that measuring the maximum MD and FL diameter of the crown was deemed impossible, were excluded. Crown and root surface morphology was reviewed by three clinicians (a specialist in reparative dentistry, an endodontic resident and a general dentist) and was divided into two groups (first premolar and second premolar) after reaching a consensus.

After immersing the teeth in 5.25% sodium hypochlorite for 15 min, the tissue tags, calculus, and bone spurs were removed by scaling and polishing the root surface.

**Figure 1.**
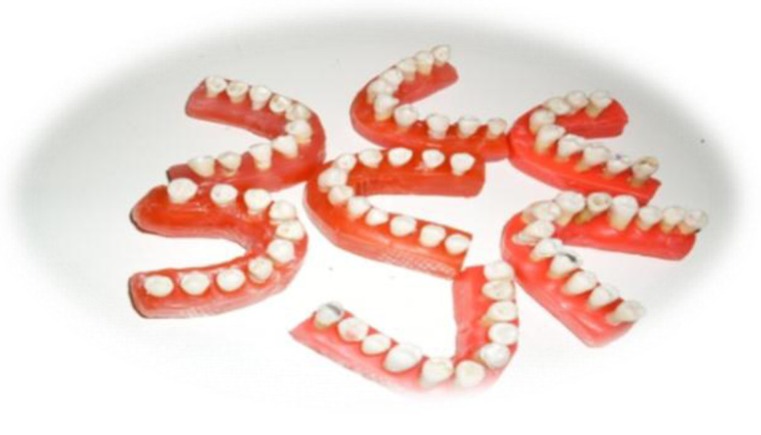
Teeth were mounted in rose wax casts in dental arch form

In order to homogenize and stabilize samples’ position, the teeth were mounted in rose wax casts in dental arch form (Figure 1). The teeth were exposed by CBCT device (Scanora 3D; Soredex, Tuusula, Finland) with 6 mA and 89 kVp. All scanners include an x-ray source and detector installed on a Gunther with 360 degrees rotation. As a result during rotation, there will be many exposures at fixed intervals creating some fixed images called *base images*. Finally, by means of these sectional images and according to the specific objectives of the plan, the evaluations were carried out. Moreover, the images were analyzed using 3D dental software. 

### A-Analysis of Root Canal System

**Figure 2.**
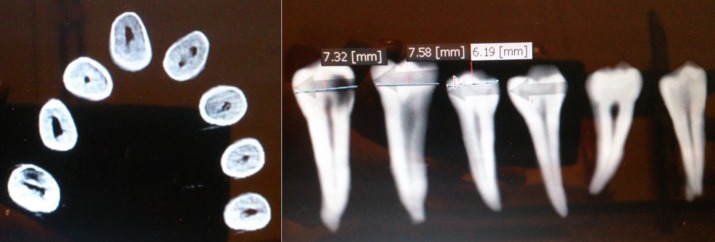
Coronal and axial sections of CBCT images of subjects

The root canal system was analyzed both in MPR and Dental programs by axial sections (Figure 2). The sections in 0.1 mm slices were analyzed serially from coronal to apical. According to the sequence of tooth sections, the configuration of canal system was obtained based on Vertucci classification. While analyzing the root canal system, in addition to ×2 filter option and changes in contrast and density, the Invert option and ×1 zoom were used to increase accuracy and also to reduce eye strain. If there was any doubt regarding the number of root canals during the analysis of axial sections, the number and type of the canals were confirmed in coronal (Figure 2) and sagittal sections. 

Before conducting the one-way ANOVA, equality of the variance of crown size variable was evaluated in different groups using Levene’s test and the normality of its distribution in different groups was also examined by Kolmogorov–Smirnov test.

### B-Crown Size

The following steps were performed to check the size of the crown: first, the arch of the cast was drawn by selecting dental option and observing the axial view. Then, by selecting the measurement option, the maximum mesiodistal diameter of each tooth was calculated separately in millimeters. Similarly, the maximum buccolingual diameter of each tooth was determined cross-sectionally. Finally, the rate of maximum mesiodistal diameter to maximum buccolingual diameter of each tooth was obtained.

## Results

From a total of 42 mandibular first premolars, 71% of the teeth were Vertucci type I and 29% were Vertucci type V, and from a total of 41 mandibular second premolars, 76% were Vertucci type I and 22% were Vertucci type V. Only one tooth (2%) showed Sert and Bayirli type IX classification; however, because only tooth types based on Vertucci classification were evaluated in this study, this tooth (type IX) was excluded from the relevant statistical analyses (Table 1). 

**Table1.** Frequency distribution of canal types inmandibular first and second premolars

Tooth Type		Canal Type (Vertucci classification)			
		I	V	IX	Total
**First Premolar**	Number (%)	30(71.4%)	12(28.8%)	0(0%)	42(10.0%)
**Second Premolar**	Number (%)	31(75.6%)	9(22%)	1(2.4%)	41(10%)

The Chi-square test showed that there was not a significant difference (*P*=0.488) between the frequency of canal types in both dental groups (mandibular first and second premolars).

The two-way analysis of variance (Two-way ANOVA) showed that tooth type (mandibular first and second premolars) affects the size of the crown (*P*=0.03) and also in the mandibular first premolar, the average size of the crown is greater than that of mandibular second premolar (Table 2), but there was not a significant relationship between crown size and canal system morphology (*P*=0.78), that is, the average size of the crown does not affect mandibular first and second premolars with multiple canals. 

**Table 2.** Mean (SE) of crown size inmandibular first and second premolars

Group of teeth	Type I Mean(SE)	Type V Mean(SE)
**First premolar**	0.87(0.023)	0.92( 0.031)
**Second premolar**	0.84(0.026)	0.78(0.096)

## Discussion

Our results concerning the number and type of the canals indicate that almost three quarters of the samples (73.5%) were single canals (type I) and a quarter (25.3%) had a type V morphology. In 1.2% of the samples, the type IX of Sert and Bayirli classification (one canal with one orifice leading to three foramina in apical part) was observed. The mentioned classification includes 14 types of extra canals that were not included in previous classifications [3]. However, because the identification of canal type in this study was based on Vertucci classification, this sample was excluded from statistical analysis.

Totally, almost 30 first premolars (71.4%) and 31 second premolars (75.6%) had single canals (type I), and 12 first (28.6%) and 9 second premolars (22%) had more than one canal (type V). Kartal and Yanikoglu reported the prevalence of mandibular premolars with single canal and mandibular premolars with more than one canal as being 72.2% and 27.8%, respectively [9], which is consistent with the present study in this regard.

With a full-mouth radiographic survey, Serman and Hasselgren found that 15.7% of mandibular first premolars and 7% of mandibular second premolars had a divided canal, which means that the number of divided canals in mandibular first premolars are greater than those in mandibular second premolars [10]. This result is consistent with the results of our study.

Zillich and Dowson found that 0-34.3% of second premolars had two or more than two canals. They also reported the prevalence of triple canal type as 0.4% [11]. These figures are almost within the same range of the data of the present study and this slight difference can be justified by the sample size and identification technique of the canals (CBCT or radiography).

By using a vulcanized rubber and histologic evaluation of stained sections, Barrett found that 18.7% of mandibular first premolars and 3.1% of mandibular second premolars had more than one canal. The results of Barrett’s study are almost similar to the results of Okumura’s study in which the prevalence of the first and second premolar teeth with more than one canal were 24% and 3.1%, respectively [12, 13].

With the total number of 16 anatomical studies evaluating only the mandibular first premolar, Ingle estimated the average prevalence of the first premolar with single canal, and two or more than one canals as being 75.8% and 24.2%, respectively [3]. This result is very close to the results of our study. It is interpreted that despite various methods and techniques used in different studies, the prevalence of two or more canals in the mandibular premolars is higher than 20%, which is a little more in mandibular first than the mandibular second premolars.

Moreover, almost all canal types identified in this study were Vertucci type V, which is consistent with the results of Yoshioka’s study. Yoshioka also introduced type IV or V (85.2%) as the most common type seen in radiographs of mandibular first premolars with more than one canal [4].

In the present study, the average crown size of the mandibular first and second premolars were 0.88 and 0.82, respectively, meaning that with statistically significant difference; the average crown size in mandibular second premolars is lower than the mandibular first premolars (*P*=0.03). This finding is in line with Ingle’s statements, but is inconsistent with Behnaz’s descriptions who supposed the crown of mandibular second premolar to be a little bulkier than the first premolar. This difference is probably due to different definitions of the size of the crown and measurement techniques in the studies mentioned earlier. In our study, the crown size was measured based on Peck & Peck index from maximum mesiodistal diameter to maximum buccolingual diameter [3, 14].

In the current study, no significant relationship was seen between the crown size and the prevalence of an extra canal in any of the mandibular first or second premolars (*P*=0.78). Although the average size of the crown in mandibular first premolars with two canals (type V) was a little higher than in the first premolars with one canal, this difference was not statistically significant, and in this regard, it is inconsistent with the results of warren and Laws’ study. They reported statistically significant linear relationship between the size of the crown and the two canals existing in mandibular incisors; that is, the more decreasing the crown size, the more probable is the existence of two canals in mandibular incisors [6]. It is likely that the differences mentioned earlier between these two studies is due to different methods of measuring index (Caliper or CBCT), method of evaluating the number and type of canals (conventional radiography against CBCT), the number and type of the studied dental groups (incisor or mandibular premolar), commentators and the incidence of bias between them.

In a case report of the first and second premolars with three canals, Nallapati stated that the cervical half of the root is often wider than usual, and these teeth often have the least tapering [1]. According to this research, seeing apparent changes in the crown of first premolars with more than two canals is not unexpected. As seen in our study, in a mandibular second premolar with 3 canals in apex (Bayirli, Sert type IX), the crown size was a little bigger than in others. However, since the incidence of mandibular premolars with three canals is rare, more examination of samples is needed to prove this.

Neelakantan *et al*. reviewed seven methods for the evaluation of root canal system, including contrast medium enhanced digital, plain digi, spiral CT, peripheral quantitative, Cone-beam CT, clearing technique, and modified canal staining. They concluded that clearing and staining is the gold standard method for the evaluation of root canal morphology and CBCT is also a valuable method applicable in both *in vitro* and *in vivo* studies[15].

Matherne *et al*. showed the priority of CBCT over Charged Coupled Device (CCD) and photo-stimulable phosphor plates, in identification of root canal system [16].

By using coloring and making transparent method, Awawdeh *et al*. evaluated the root form and canal morphology of mandibular premolars. They reported the prevalence of mandibular premolars with a single canal (type I) as 72% and the prevalence of two canals with two separate foramina as 23% [17], which is consistent with the results of our study. This means that, compared to coloring and making transparent method, CBCT is considered as a reliable, accurate, and yet competitive method which is attracting attention.

Considering apparent relationship between the crown and the prevalence of three canals in the first mandibular premolars and the rare prevalence of this type of canal in general, another investigation with larger sample size to examine also the same hypothesis is suggested.

Conducting a similar study but with another definition for crown size *e.g*. its length or its cervical width is recommended, in order to resolve any doubt concerning this subject.
